# The Prevalence and Associated Factors of Depressive Symptoms Among Medical Students in Bangladesh During the COVID-19 Pandemic: A Cross-Sectional Pilot Study

**DOI:** 10.3389/fpubh.2021.811345

**Published:** 2022-01-31

**Authors:** Md. Abdullah Al Jubayer Biswas, M. Tasdik Hasan, Nora Samir, Sayma Islam Alin, Nusrat Homaira, Md. Zakiul Hassan, Mst Rabeya Khatun, Afifa Anjum, Sahadat Hossain, Kamrun Nahar Koly, Farhana Safa, Syeda Fatema Alam, Md. Abdur Rafi, Md. Abdullah al Osman Biswas, Farida Yasmin, Vivek Podder, Tonima Islam Trisa, Dewan Tasnia Azad, Rhedeya Nury Nodi, Fatema Ashraf, S. M. Quamrul Akther, Helal Uddin Ahmed

**Affiliations:** ^1^Department of Statistics, University of Dhaka, Dhaka, Bangladesh; ^2^Public Health Foundation, Bangladesh (PHF, BD), Dhaka, Bangladesh; ^3^Department of Public Health, State University of Bangladesh (SUB), Dhaka, Bangladesh; ^4^Department of Primary Care and Mental Health, University of Liverpool, Liverpool, United Kingdom; ^5^Discipline of Paediatrics, School of Women's and Children's Health, The University of New South Wales, Sydney, NSW, Australia; ^6^Department of Public Health and Informatics, Jahangirnagar University, Dhaka, Bangladesh; ^7^International Centre for Diarrheal Diseases Research, Bangladesh (icddr, b), Dhaka, Bangladesh; ^8^Department of Biotechnology and Genetic Engineering, Mawlana Bhashani Science and Technology University, Tangail, Bangladesh; ^9^Department of Public Health, Dalla Lana School of Public Health, University of Toronto, Toronto, ON, Canada; ^10^Shaheed Suhrawardy Medical College Hospital, Dhaka, Bangladesh; ^11^Rajshahi Medical College, Rajshahi, Bangladesh; ^12^Sheikh Sayera Khatun Medical College, Gopalgonj, Bangladesh; ^13^Tairunnessa Memorial Medical College and Hospital, Gazipur, Bangladesh; ^14^Jashore Medical College, Jashore, Bangladesh; ^15^National Institute of Mental Health, Dhaka, Bangladesh

**Keywords:** medical students, depressive symptoms, patient health questionnaire-(PHQ-9), COVID-19, Bangladesh

## Abstract

**Background:**

Whilst very limited studies have demonstrated a correlation between the COVID-19 pandemic and depressive symptoms amongst Bangladeshi medical students, the prevalence and associated factors of depressive symptoms as measured by the Patient Health Questionnaire (PHQ-9) remains widely unknown.

**Objective:**

The study aimed to investigate the prevalence and factors associated with depression symptoms among Bangladeshi medical students during the COVID-19 pandemic lockdown period.

**Method:**

In this web-based cross-sectional pilot study, medical students' data was collected using the Google Forms web survey platform after obtaining electronic informed consent. A total of 425 medical students were selected using a systematic sampling technique to accumulate depression symptoms and demographic and pandemic-related information. Depression was measured by a self-administered, validated English version of the Patient Health Questionnaire (PHQ-9) tool. The descriptive analysis utilized frequency and percentages, while the stepwise binary logistic regression analysis was performed to investigate the factors associated with depressive symptoms.

**Result:**

Among 425 medical students, 62.3% were female, 97.4% unmarried. Almost 80.2% of medical students had mild to severe levels of depressive symptoms as characterized by PHQ-9. A significantly higher probability of depression was found amongst female students (adjusted OR = 1.8), those who struggled to stay away from social media (adjusted OR = 1.8), those who tried to be optimistic for maintaining better psychology (adjusted OR = 11.1), and those who always had a sleeping difficulty in the last 4 weeks (adjusted OR = 8.9).

**Conclusion:**

A very high prevalence of depression symptoms among Bangladeshi medical students was found across the majority of socio-demographic variables. The alarming prevalence and associated factors of depression suggests the need for follow-intensity psychosocial interventions designed for medical students during the COVID-19 pandemic

## Introduction

As of December 23, 2021 the COVID-19 crisis has overwhelmed healthcare systems worldwide and resulted in over 5.3 million deaths and 273 million infections (1). The mental health and well-being of health care workers have been particularly impacted during the COVID-19 outbreak, with an increased prevalence of anxiety, fear, depression, and insomnia reported. Reasons for higher anxiety and depressive symptoms reported by health care workers during the pandemic include extended work shifts, higher risk of infection, lack of adequate personal protective equipment (PPE) and prolonged separation and isolation from families and friends (2). Medical students, in particular, are at risk of developing adverse mental health outcomes due to changes in teaching techniques, interruptions in academic curricula and clinical rotations, increased workload, and viral exposure during the COVID-19 epidemic (3–5). A meta-analysis found that the COVID-19 pandemic had a substantial adverse effect on the mental well-being of medical students (6). In addition, psychological reactions and depressive symptoms have been intensified in various other contexts due to COVID-19 pandemics (7, 8). During the COVID-19 pandemic in Brazil, 64.4% of medical students reported depressed symptoms using the Patient Health Questionnaire (PHQ-9), whereas, in India, it was 44.89% using the DASS-21 (5, 9).

Like the general population, medical students in Bangladesh have been demonstrated to suffer detrimental psychological impacts due to the COVID-19 epidemic (10, 11). A cross-sectional study during the COVID-19 pandemic reported that 49.9% of 425 Bangladeshi medical students had depressive symptoms measured by the Hospital Anxiety & Depression Scale (HADS) (10). However, there are limited data on prevalence and the associated factors of depressive symptoms using PHQ-9 during the COVID- 19 pandemic medical students. Additionally, it's unknown how the social isolation during lockdown periods in Bangladesh impacted the prevalence of depressive symptoms amongst Bangladeshi medical students. Also, in light of the long-term psychological effects of COVID-19, Bangladeshi medical students' depression status needs to be assessed so that an appropriate mitigation strategy may be devised in the future. Therefore, our study aimed to assess the prevalence and factors associated with depressive symptoms among medical students using PHQ tools during the COVID-19 pandemic lockdown period in Bangladesh. We also hypothesized that the prevalence of depression among Bangladeshi medical students would be the same regardless of their demographics or any other information about the epidemic. The findings of this study may help educational stakeholders understand medical students' mental status during health crises and plan targeted interventions to address such issues in the present pandemic and for future public health crises.

## Methodology

### Study Setting and Population

An online cross-sectional pilot survey was conducted between April 21, 2020, and May 10, 2020, to explore prevalence and factors associated with depressive symptoms among Bangladeshi medical students, coinciding with the 1st wave of the COVID-19 pandemic. All medical students who were Bangladeshi citizens, aged ≥18 years, currently enrolled in undergraduate medical program (MBBS) in any Bangladeshi medical college, residing in Bangladesh during the pandemic, had access to the social media platforms including Facebook, WhatsApp, Twitter or an e-mail account, and could read and understand English were eligible to participate.

### Data Collection

We designed an online survey data collection tool with the declaration of anonymity and confidentiality using the Google Forms web survey platform to minimize human contact and adhere to the strict COVID-19 protocols. Initially, we recruited five volunteer medical students conveniently from five different medical colleges situated in different locations in Bangladesh, including Chittagong, Dhaka, Sylhet, Barisal, Rajshahi. The five volunteers developed a primary contact list of medical students using their social media platforms, such as Facebook, WhatsApp, and Twitter. After finalizing the primary contact list, the study team selected medical students from the list and sent an invitation message with a link for the survey using given e-mails or social media profiles. The invitation letter explained the rationale, objectives, and nature of the project. Medical students who accepted the invitation provided their responses by browsing the link; otherwise, they were counted as non-response.

### Depressive Symptoms Measure

A self-administered version of the Patient Health Questionnaire (PHQ-9), the PRIME-MD diagnostic instrument for measuring depression, was utilized to assess depression symptoms (12). An English version of nine items PHQ-9 depression module whose reliability and validity have been reported by multiple studies was designed on the Google Form platform (13). A four-point Likert scale layout was followed to create an online PHQ-9 section where each item of the PHQ-9 scale was scored from zero implied not problematic at all to three indicated extremely difficult. The global summation of the nine issues delineated the level of the severity of depression. Recommended cut off PHQ-9 scores for level of depression severity (12): minimal (score 0–4), mild (score 5–9), moderate (score 10–14), moderately severe (score 15–19), severe (score 20–27). Patient Health Questionnaires had good internal consistency (Cronbach's Alpha = 0.77), adequate split-half reliability (*r* = 0.80) in our data.

### Demographic and Pandemic Related Information

The self-reported and structured demographic and pandemic related questionnaire had five sections: socio-demographic, tension related to COVID-19 infection, adherence with media, the strategy taken to maintain psychological health and difficulty in sleeping. Participants filled a brief section after the informed consent segment on demographic characteristics including age in year, gender, marital status, profession, monthly income, ever searching remedy for mental health. In the next susceptible to COVID-19 section, participants invited the questions related to tension about himself/herself and family members getting infected by COVID 19, hard to step ways from media. Also, to evaluate respondents' recreational activities, they were asked questions regarding leisure activities, time to spend on leisure activities, and struggling to stay away from media. Furthermore, the difficulty in sleeping cycles was assessed using questions related to sleeping disturbance faced in the last 4 weeks and the average sleep time during the previous 4 weeks.

### Sample Size and Sampling Technique

**Figure 1:**
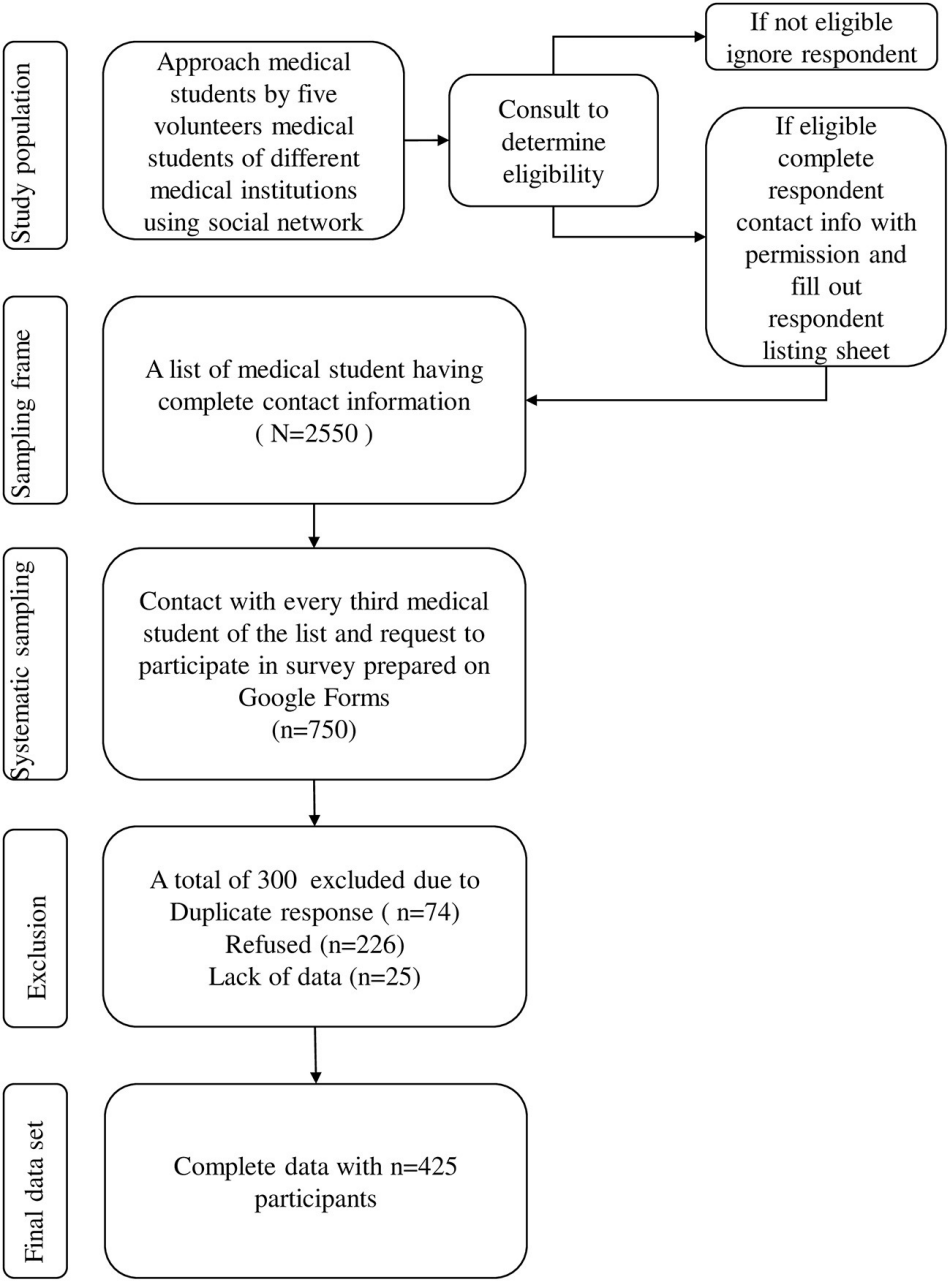
Flow chart of sampling strategy.

We calculated sample size using a single population proportion formula and considering 74.4% mild to severe depression assessed by the Patient Health Questionnaire (PHQ-9) among medical students of Banaras Hindi University, Varanasi, Uttar Pradesh, India (14, 15). Considering a 95% confidence interval (CI), 5% absolute precision, 5% non-response rates, and a 1.27 design effect, a minimum sample of 390 was calculated. A systematic sampling technique was used where every third eligible medical student was selected and approached to participate in the study. The final contact list was used as a list-based sampling frame (16, 17). The detailed sampling strategy is shown in Figure 1.

### Statistical Analysis

We conducted descriptive analyses using frequency, percentage, mean, and standard deviation (SD) depending on the variables' type. Depressive symptoms of the study participants were categorized using established cutoff and summarized using frequency and percentage (12). Cross-tabulation with Pearson's Chi-square was used to test the association of demographic and pandemic related variables with participants' severity of the depressive symptom. We executed a binary logistic regression analysis to explore the bivariable relationship between the respondent's depression symptoms and explanatory variables. We reported bivariable analysis output as the unadjusted odds ratio (UOR) with a 95% confidence interval. We utilized stepwise logistics regression with removal algorithm to identify the factors associated with depression and described as adjusted odds ratio (AOR) with 95 % confidence interval (14, 15) for multivariable analysis. We included variables in the final multivariable model, which were significant at the 5% significance level. Statistical significance of the association was considered for *p*-values < 0.05. The analysis was performed using Stata software (Stata Corp. 2017. Stata Statistical Software: Release 13. College Station, TX: Stata Corp LP).

### Ethical Consideration

The study received ethical approval from the Ethical Review Committee, Shaheed Suhrawardy Medical College, and Dhaka, Bangladesh (ShSMC/Ethical/2020/12). A concise outline of the study and information regarding ethics were provided on the google form's preliminary page. Confidentiality of the participants was strictly maintained by avoiding identifiable personal questions, and data was collected anonymously. The respondents were also informed about their voluntary participation and ending the survey at any time just by closing the web browser. Likewise, the consent field was kept as a mandatory field for starting the study. The study was carried out under the Checklist for Reporting Results of Internet E-Surveys (CHERRIES) guideline (18). Furthermore, the study investigators monitored all procedures relevant to the study to ensure the proper ethical standards of the concerned national and institutional committees on human experimentation and the Helsinki Declaration of 1975, as revised in 2008.

## Results

The final contact list had 1,368 medical student contact information, and among them, 456 medical students were identified and sent the invitation. After excluding 31 responses due to duplicate response, lack of complete records, the data set of 425 responses were finalized for analysis. This study had a response rate of around 93.2%.

### Demographic and Pandemic Related Information

Among 425 medical students, 62.3% were female, and the mean age was 22 years with a standard deviation of 1.8 years. Almost all the students were fully engaged with the study (87.5%) and never sought treatment for their mental health issue (93.2%) (Table 1).

**Table 1:** Prevalence of mild to severe depressive symptom among medical students, measured by PHQ-9, during COVID-19 pandemic following their demographic and pandemic related characteristics, 2020 Bangladesh.

**Variables**		**Prevalence of depression**		
	**% (*n*)**	**% (row)**	**95% CI**	***P*-value**
**Among all participants**	100.0 (425)	80.2	(76.1, 83.9)	
**Age in year**	22.0 ± 1.8			
≤ 20	25.4 (108)	72.2	(63.0, 79.9)	
21–24	68.5 (291)	83.5	(78.8, 87.4)	0.038
≥25	6.1 (26)	76.9	(56.7, 89.4)	
**Gender**				
Male	37.7 (160)	74.4	(67.0, 80.6)	
Female	62.3 (265)	83.8	(78.8, 87.8)	0.018
**Marital status**				
Married	2.6 (11)	90.9	(53.5, 98.8)	0.368
Unmarried	97.4 (414)	79.9	(75.8, 83.5)	
**Profession**				
Part-time job	12.5 (53)	90.6	(79.1, 96.1)	0.044
Solely study	87.5 (372)	78.8	(74.3, 82.6)	
**Ever seeking treatment for mental health issues**				
Yes	6.8 (29)	89.7	(71.9, 96.7)	0.187
No	93.2 (396)	79.5	(75.3, 83.2)	
**The tenseness of getting infected by COVID-19 about**				
**Himself/herself**				
Severe	36.9 (157)	84.1	(77.4, 89.0)	
Moderate	43.5 (185)	79.5	(73.0, 85.0)	0.209
No/minimal	19.5 (83)	74.7	(64.2, 83.0)	
**Family members**				
Severe	64.2 (273)	84.3	(79.4, 77.8)	
Moderate	24.0 (102)	76.5	(67.2, 83.7)	0.006
No/minimal	11.8 (50)	66.0	(51.8, 77.8)	
**Source of news**				
Television news	68.5 (291)	80.8	(75.8, 84.9)	
Social media	18.8 (80)	86.3	(76.7, 92.3)	0.038
Newspaper	12.7 (54)	68.5	(54.9, 79.6)	
**Struggling to get away from social media**				
Yes	71.1 (302)	83.1	(78.4, 87.0)	0.020
No	28.9 (123)	73.2	(64.6, 80.3)	
**The strategy took to maintain healthy psychology**				
Yes	44.2 (188)	80.8	(74.6, 85.9)	
No	55.8 (237)	79.7	(74.0, 84.3)	0.760
**Type of strategic strategy taken to maintain healthy psychology (Multiple responses)**				
Involving leisure activities	55.3 (104)	80.8	(72.0, 87.4)	0.832
Spending quality of time with friends and family	29.3 (55)	87.3	(75.4, 93.9)	0.160
Maintaining COVID-19 instructions	21.8 (41)	73.2	(57.5, 84.6)	0.232
Practicing religion norms	21.3 (40)	80.0	(64.6, 89.7)	0.969
Optimistic thinking/positive outlook	17.6 (33)	96.9	(80.1, 99.5)	0.012
Maintaining physical activity	17.0 (32)	90.6	(74.2, 97.0)	0.125
Staying at home	7.5 (14)	71.4	(42.8, 89.3)	0.400
Avoiding COVID-9 new broadcast	2.1 (4)	80.0	(25.5, 97.9)	0.989
**Difficulty in sleeping**				
**Having sleeping disorder in last 4 weeks**				
Always	10.8 (46)	93.5	(81.4, 97.8)	
Often	14.8 (63)	92.1	(82.1, 96.6)	
Sometimes	24.5 (104)	87.5	(79.6, 92.6)	<0.001
Occasionally	22.8 (970)	86.6	(78.2, 92.1)	
Never	27.1 (115)	56.5	(47.3, 65.3)	
**Average time of sleep in last 4 weeks**				
<6 h	19.8 (84)	83.3	(73.7, 89.9)	
6–8 h	36.0 (153)	75.8	(68.4, 81.9)	0.226
More than 8 h	44.2 (188)	82.4	(76.3, 87.3)	

### Prevalence of Depressive Symptoms Among Medical Student

**Figure 2:**
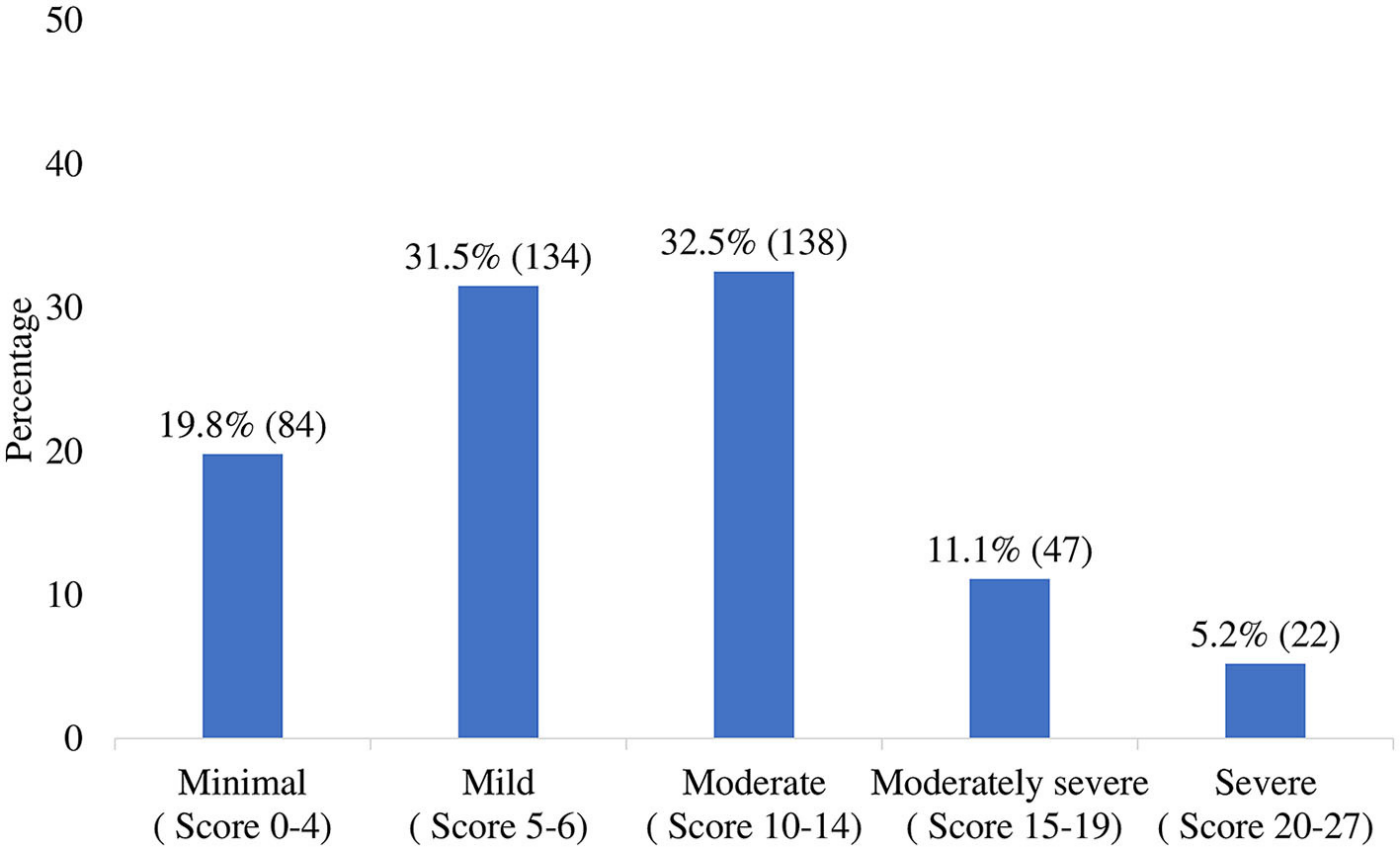
Level of depression severity among medical students obtained by categorizing PHQ-9 score. The total PHQ-9 score was 4,038, with an average 9.5 ± 5.4 and a median 9.0.

Among all study participants, the average PHQ-9 score was 9.5 with a standard deviation of 5.4 and a range between zero to 26 (Figure 2). The prevalence of mild to severe depressive symptoms was 80.2% where it was high among females (83.8%) and among married students (90.9%). Likewise, the prevalence was decreased significantly with decreasing tension about the family member getting infected by COVID-19, ranging from 66.0 to 84.3%. Moreover, the significant highest prevalence was observed for the medical student who struggled to get away from social media (83.1%), always faced sleeping disturbances in the last 4 weeks (93.5%), then counters category (Table 1).

### Associated Factors of Depressive Symptoms Among Medical Students

Table 2 shows the outcome of the bivariable and multivariable analyses. After controlling for other factors, the multivariable analysis found a higher probability of depression symptoms among female medical students (AOR = 1.8). Additionally, depressive symptoms remained almost similar among four successive categories of the respondents who had a sleeping disturbance in the last 4 weeks (Always vs. Never AOR = 8.9, 95% CI: 2.6–31.4; Often vs. Never AOR = 7.9, 95% CI: 2.8–21.7; Sometimes vs. Never AOR = 5.6, 95% CI: 2.7–11.5; Occasionally vs. Never AOR = 5.0, 95% CI: 2.3–9.7). Also, students who maintained a positive outlook for keeping psychological health fit had a higher probability of being depressed during the COVID-19 pandemic (AOR = 11.1).

**Table 2:** Logistic regression analysis of medical student who had depression for mild to severe level during COVID-19 pandemic, 2020 Bangladesh.

	**Depression (mild to severe level)**			
	**Unadjusted OR (95% CI)**	***P*-value**	**Adjusted OR (95% CI)**	***P*-value**
**Age in year**				
21–24	1.9 (1.2–3.3)	0.012	–	
≥25	1.3 (0.5–3.5)	0.628	–	
≤ 20	Reference			
**Gender**				
Female	1.8 (1.1–2.9)	0.019	1.8 (1.1–3.1)	0.032
Male	Reference		Reference	
**Profession**				
Parttime job	2.6 (1.0–6.7)	0.051	–	
Solely study	Reference			
**The tenseness of getting infected by COVID-19 about family members**				
Severe	2.8 (1.4–5.4)	0.003	–	–
Moderate	1.7 (0.8–3.5)	0.174	–	–
No/minimal	Reference			
**Adherence with media**				
**Source of news**				
Television news	2.9 (1.2–6.8)	0.016	–	–
Social media	1.9 (1.0–3.7)	0.046	–	–
Newspaper	Reference			
**Struggling to get away from social media**				
Yes	1.8 (1.1–3.0)	0.021	1.8 (1.0–3.1)	0.041
No	Reference		Reference	
**Strategy took to maintain psychological health**				
**Type of strategic capture**				
Optimistic thinking/positive outlook				
Yes	8.6 (1.2–63.8)	0.035	11.1 (1.3–93.5)	0.034
No	Reference		Reference	
**Difficulty in sleeping**				
**Having a sleeping disorder in the last 4 weeks**				
Always	11.0 (3.2–37.6)	<0.001	8.9 (2.5–31.4)	0.001
Often	8.9 (3.3–23.9)	<0.001	7.9 (2.8–21.7)	<0.001
Sometimes	5.4 (2.7–10.7)	<0.001	5.6 (2.7–11.5)	<0.001
Occasionally	5.0 (2.5–9.9)	<0.001	4.9 (2.3–9.8)	<0.001
Never	Reference		Reference	
**Average time of sleep in the last 4 weeks**				
6–8 h	0.6 (0.3–1.2)	0.180	–	
More than 8 h	0.9 (0.5–1.9)	0.858	–	
<6 h	Reference			

## Discussion

Our study aimed to determine the prevalence and factors associated with depressive symptoms among Bangladeshi medical students during the COVID-19 pandemic. The study findings revealed that the prevalence of mild to severe depressive symptoms was high in medical students, and factors such as gender, struggling to get away from social media, and having sleep disturbances in the preceding 4 weeks were significantly associated with depressive symptoms.

In our study, 80.2% of medical students had mild to severe levels of depressive symptoms, which was comparable to findings from Bangladesh (49.1%), India (74.6%) and Brazil (64.41%) but higher than those reported from Nepal (5.5%) and Iran (25.6%) (3, 3, 5, 10, 19, 20). The disparity in prevalence could be due to the usage of multiple measurement scales and countries contexts. Additionally, the tension associated with the possibility of infecting a family member with COVID-19, gender, adverse effects of COVID-19 and its perceived long-term health outcomes, discrimination against the frontline physicians and a tendency to get irritated more quickly than normal could all contribute to the high prevalence (10, 21). The study by Tasdik et al. reported depression symptoms in 38.9% of medical students, with 3.6, 14.5, and 20.8% being severe, moderate, and mild depression, respectively pre-COVID-19 era, which used PHQ-9 as the assessment tool. This highlights the overwhelming mental health burden experienced by the medical students during the pandemic (22).

Our study found that female medical students reported experiencing significantly more depressive symptoms than male students, comparable with earlier epidemiological studies (10, 23). Research on the disparity between women and men during the COVID-19 pandemic revealed that female students had higher COVID-19 pandemic risk perceptions than male students (23, 24). That research also estimated higher conscientiousness, neuroticism, tolerance to experiences, and tension to be higher in female university students (23). However, in comparison to results from a similar COVID-19 pandemic survey, it was found that gender did not significantly affect the medical students' mental health (25). In light of our study, further investigation into understanding the kinds of social support that can help mitigate gender-specific mental health well-being issues among Bangladeshi medical students is essential.

Additionally, we also found that medical students who fail to disengage from social media during the COVID-19 pandemic tend to experience more frequent depressive symptoms. At the height of the COVID 19 pandemic, students were unable to leave their homes for fear of being infected or breaking government-imposed lock-down laws (26). Online platforms were initially used to learn about the virus and spread information, which resulted in a spike in mobile social media use (26). Maintaining social media use for an extended period may cause social, family, and/or occupational impairments, cyberchondria as well as mental health and well-being problems (26–28). A recent survey of 100 first-year medical students in India showed that time spent on social media for over 4 hours during lock-down rose from 1.1 to 47.72% (29). It was also found that social network use of >4 h is significantly correlated with mood variations, including feeling frustrated among medical students (29). Based on our findings, we believe institutions and clinicians must work together to find ways to combat social media addiction among medical students and encourage healthy use of social media during the pandemic. In order to get a clearer understanding of how medical students should utilize social networking channels as helpful learning resources, further research is needed.

We also found that medical students who had a sleeping disorder in the last 4 weeks were more likely to have depressive symptoms, similar to a previous prospective longitudinal study conducted in India on 217 medical students (30). In that study, researchers found that medical students who had increased depression during the COVID-19 pandemic were 1.11 times more likely to have poor sleep quality (30). Because of travel limitations and lock-down precautions, medical students were dealing with reduced physical activity, changing living circumstances, and greater employment pressure (30). Sleep was adversely affected by these combinations, one of the key symptoms of seeking depression (30, 31). In addition, it may highlight the need for the medical community to provide further support to medical trainees at times of health crises such as the COVID-19 pandemic in order to prevent sleep disorders, burnout and associated downs-stream psychological effects.

### Strength and Limitation

It was one of the first few studies to examine the prevalence and associated factors of depressive symptoms among medical students under lock-down scenarios, using a validated method for detecting depressive symptoms. To avoid sampling bias, we constructed a contact list of medical students based on the eligibility criteria, which also ensured representation of the population we wanted to study. However, our study has several limitations. Firstly, as we prepared a primary contact list based on five volunteer medical studnets social media networks, there might have been some selection bias in the list. Secondly, students without internet or social media accounts were excluded due to the online approach of the survey platform. As a result, our results were not generalisable to all Bangladeshi medical students. Thirdly, depressive symptoms were assessed only by self-report, which may not be consistent with professional mental health diagnoses. However, the questionnaire used has been validated for use in self-reported depressive symptoms (12). Fourthly, findings from this research do not give a comprehensive picture of COVID-19's long-term impact on depression symptoms, preventative measures, and coping techniques. Furthermore, future longitudinal studies are required to examine the ramifications of COVID-19 on the medical student's psychological well-being.

## Conclusion

We conclude that during the worldwide pandemic of COVID-19, the prevalence of depressive symptoms was alarmingly high among Bangladeshi medical students, which indicates medical students were at high risk of developing depressive symptoms during the ongoing pandemic. Given that medical students are prone to developing depression during the COVID-19 pandemic, adequate mental health services focusing on depression for students might be considered by medical colleges. Besides, in times of infectious disease outbreaks like COVID-19, when mental health issues like depression symptoms impacts academic performance, physical health, psychological well-being, interventions targeted to improve mental health conditions in medical students are crucial.

## Data Availability Statement

The study's original contributions are provided in the article/supplementary material. Any further questions should be addressed to the corresponding author.

## Ethics Statement

The study received ethical approval from the Ethical Review Committee, Shaheed Suhrawardy Medical College, and Dhaka, Bangladesh (ShSMC/Ethical/2020/12). A concise outline of the study and information regarding ethics were provided on the google form's preliminary page. Confidentiality of the participants was strictly maintained by avoiding identifiable personal questions, and data was collected anonymously. The respondents were also informed about their voluntary participation and ending the survey at any time just by closing the web browser. Likewise, the consent field was kept as a mandatory field for starting the study. The study was carried out under the Checklist for Reporting Results of Internet E-Surveys (CHERRIES) guideline (Eysenbach, 2004). Furthermore, the study investigators monitored all procedures relevant to the study to ensure the proper ethical standards of the concerned national and institutional committees on human experimentation and the Helsinki Declaration of 1975, as revised in 2008. The patients/participants provided their written informed consent to participate in this study.

## Author Contributions

MTH, SFA, MAR, VP, SMQA, and FA: conceptualization. MAAJB and MRK: data analysis. MTH, SFA, MAR, VP, DA, and RNN: investigation. MAAJB, MTH, and SH: methodology. MTH, FS, SFA, and SH: resources. SMQA and FA: supervision. MAAJB, SIA, MTH, and MRK: writing –original draft. MAAJB, MTH, NS, SIA, NH, MZH, SH, FY, MAaOB, FS, KNK, SFA, MAR, VP, TIT, RNN, DA, MRK, FA, SMQA, and HUA: writing –review and editing. All authors contributed to the article and approved the submitted version.

## Conflict of Interest

The authors declare that the research was conducted in the absence of any commercial or financial relationships that could be construed as a potential conflict of interest.

## Publisher's Note

All claims expressed in this article are solely those of the authors and do not necessarily represent those of their affiliated organizations, or those of the publisher, the editors and the reviewers. Any product that may be evaluated in this article, or claim that may be made by its manufacturer, is not guaranteed or endorsed by the publisher.
